# A rare atypical rapidly involuting congenital hemangioma combined with vascular malformation in the upper limb

**DOI:** 10.1186/s12957-016-0993-3

**Published:** 2016-08-26

**Authors:** Hui Lu, Qiang Chen, Hui Shen, Ganmin Ye

**Affiliations:** 1 Department of Hand Surgery, The First Affiliated Hospital, College of Medicine, Zhejiang University, #79 Qingchun Road, Hangzhou, Zhejiang Province 310003 People’s Republic of China; 2 Departments of Orthopedics, Sanmen People’s Hospital, #171 Renmin Road, Taizhou, Zhejiang Province 317100 People’s Republic of China

**Keywords:** Rapidly involuting congenital hemangioma, Amputation, Recurrence, Capillary-lymphatic-venous malformation

## Abstract

**Background:**

Rapidly involuting congenital hemangioma is a congenital soft tissue tumor, which is difficult to excise completely and rather prone to recur. This atypical tumor combined with capillary-lymphatic-venous malformation was not reported in the literature.

**Case presentation:**

We report an atypical case of a 16-year-old teenager who was born with a mass in his right upper limb. Since there is a recurrence after excision for several times and had a serious impact on daily life, we chose amputation. After the surgery, the patient gained a functional recovery. Two years after the surgery, he had no tumor recurrence.

**Conclusions:**

For this rare tumor with repeated recurrences and significant impact on daily life, we suggest performing amputation at the early stage.

## Background

Rapidly involuting congenital hemangioma (RICH) is an uncommon, often high-flow vascular tumor that presents at birth. It is generally considered as a congenital condition [1]. Because it often invades the surrounding tissues, it is difficult to excise the tumor completely and it easily has a local recurrence [2–4]. However, surgery is still a conventional treatment for this disease. Aggressive partial resections will easily stimulate the development of the tumor, so we are supposed to avoid it in the future.

## Case presentation

**Fig. 1:**
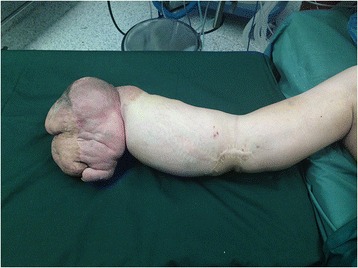
It shows that the patient had a mass on the right upper limb with movement limitation before the amputation

**Fig. 2:**
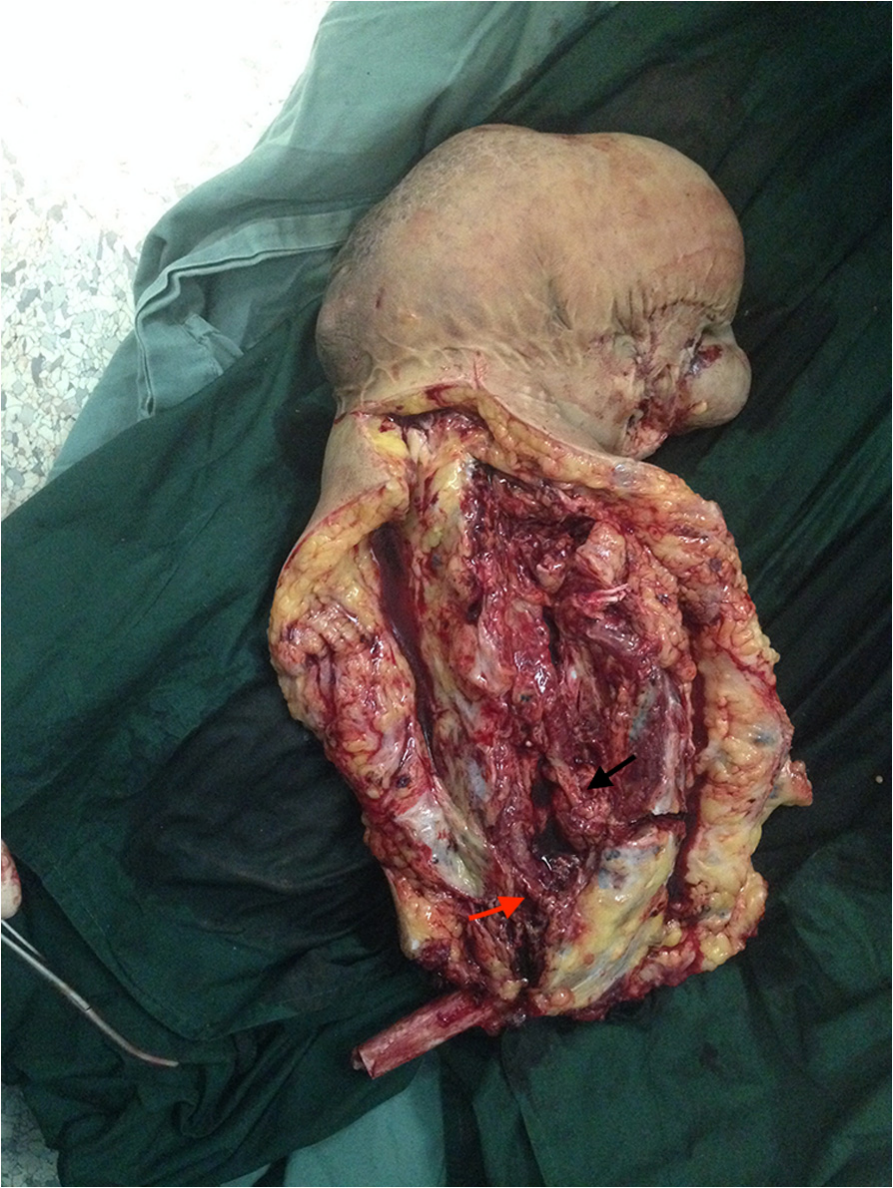
The photograph during the operation, showing segments of the skeletal muscle (*black arrow*), containing ill-defined vascular lesions and the partial thrombosis (*red arrow*)

**Fig. 3:**
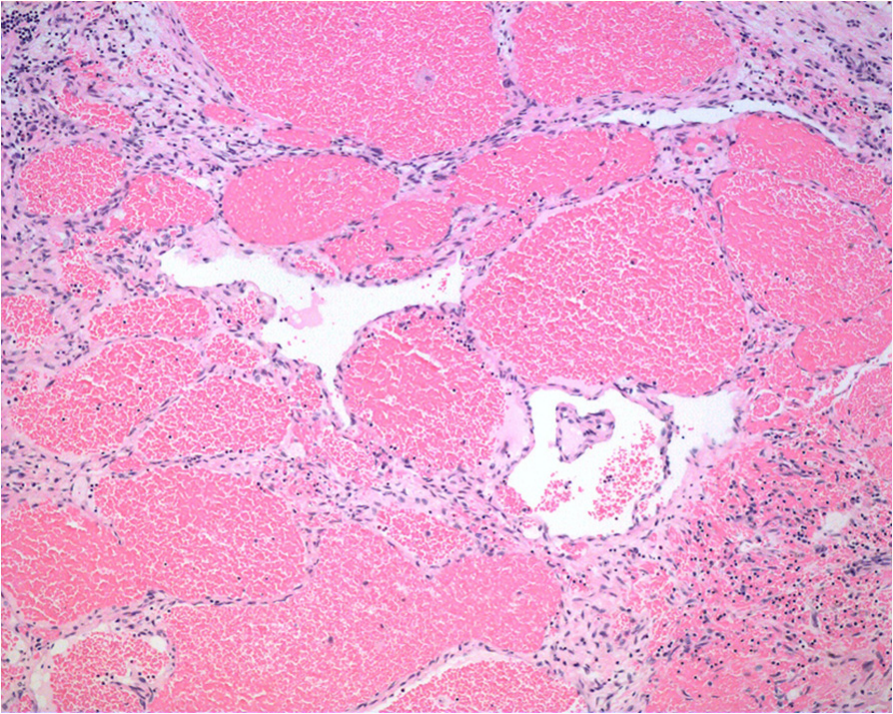
Pathology showed: skin chronic inflammation was involved with hyperpigmentation below the basal layer and thin-walled vessels (hematoxylin-eosin stain, original magnification ×100)

**Fig. 4:**
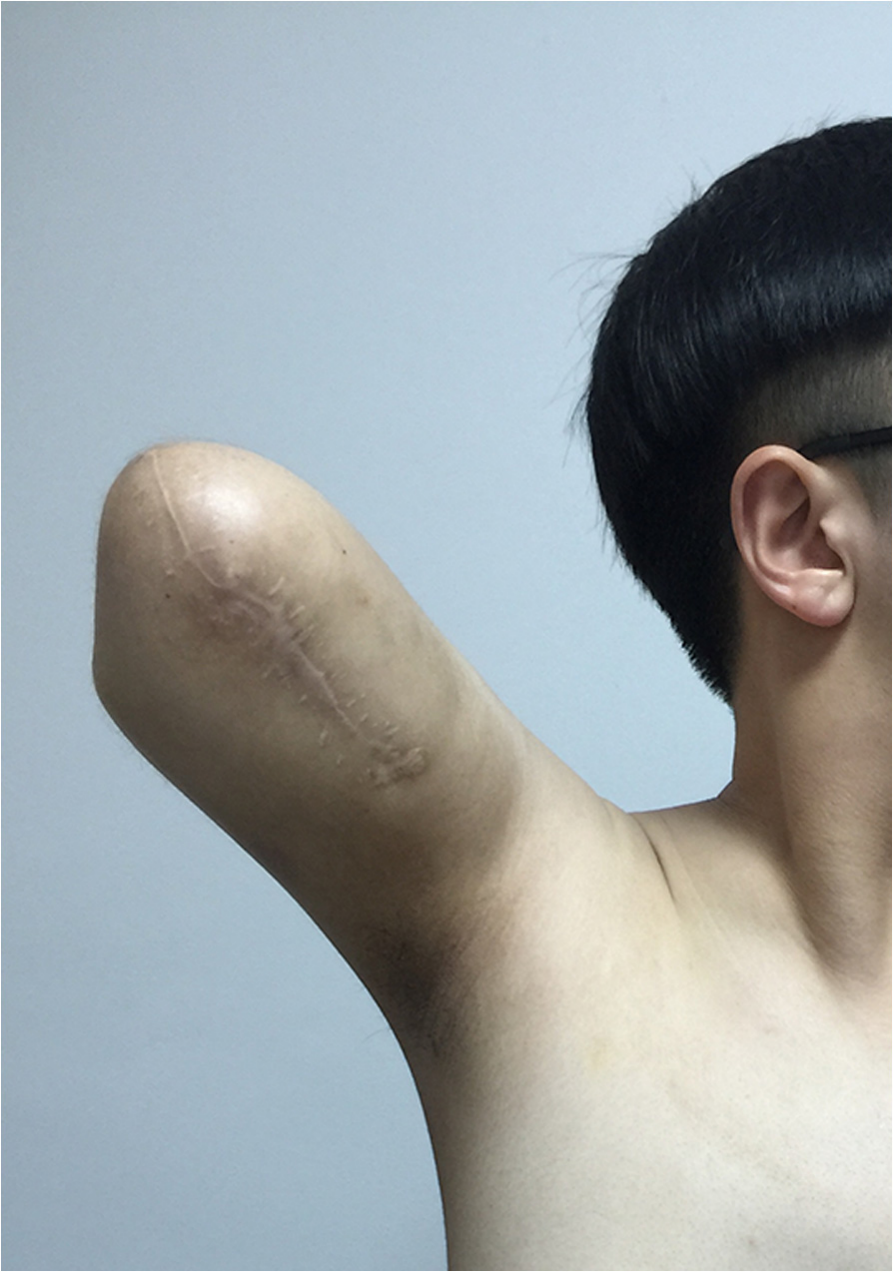
It shows that the patient was able to perform daily activities

A 16-year-old teenager who was born with a mass in his right hand, which was diagnosed as hemangioma. When he was 5 months old, the tumor was partially excised in Shanghai. The mass developed increasingly after the surgery and presented diffuse growth from the distal to proximal upper limb. The patient was treated with sclerotherapy at the age of three in Zhengzhou but the treatment failed. He was treated with excision again and abdominal pedicle flap surgery at the age of seven in Beijing. One year later, his fingers were separated. And the tumor had been partially excised for several times in Wenzhou before the patient came to our hospital. The patient’s right upper limb could not move upward. He was almost helpless in his daily life and not able to go to school. The physical examination showed multiple scars on his right upper limb and a huge mass in his right hand (Fig. 1). There was no range of motion in his right wrist and right elbow. The right shoulder muscles had atrophied. Laboratory studies revealed that hemoglobin, white cell count, and platelet count are within normal range. Erythrocyte sedimentation rate (ESR), high-sensitivity c-reactive protein, and tumor biological markers were normal. Considering the multiple recurrences of the tumor, the poor quality of his life, and the resistance to surgery, we chose amputation. Considering the patient’s need of prosthetics, the amputation was performed on the middle of the upper limb under general anesthesia. The vascular tumors and thrombosis were visible during the operation (Fig. 2). Gross examination demonstrated segments of the skeletal muscle, containing ill-defined vascular lesions, and partial thrombosis. Pathology showed that skin chronic inflammation was involved with hyperpigmentation below the basal layer and with thin-walled vessels and thin sinusoidal vascular channels (Fig. 3). Immunohistochemistry shows D2-40 (positive) and Glut-1 (negative). The diagnosis of atypical rapidly involuting congenital hemangioma (RICH) combined with capillary-lymphatic-venous malformation (CLVM) was made (ISSVA classification [5]) according to the clinical and histopathologic manifestations. The patient could raise his shoulder and use prosthetic limbs after surgery; the ability of self-care was obviously improved. He could go to school after the surgery. There was no evidence of recurrence at the 2-year follow-up (Fig. 4).

### Discussion

In this case, we had difficulty in deciding whether the tumor should be one type within the ISSVA classification. Infantile hemangioma is the commonest tumor in infants with progressive proliferation in the first year of life and slow regression in the next few years. There are also few other rare congenital hemangiomas (RICH, NICH, PICH) with different clinical courses [6–8]. Clinical course and intraoperative view suggest rare locally aggressive vascular tumor (papillary intralymphatic angioendothelioma [9]), lymphatic malformation with fat tissue component, and hemorrhage or mixed vascular malformation: lymphatic and venous (LM-VM) [10]. Venous malformations are the second malformations in occurrence after capillary malformations. None of them fits to describe the case. Klippel-Trenaunay syndrome also should be considered where two vascular malformations and soft tissue hypertrophy is present, but this patient is lack of port-wine stain and varicose veins [11, 12]. The test of Glut-1 [13] was negative; we can exclude infantile hemangioma. This patient was characterized by locally aggressive nature and the test of D2-40 was positive, tend to diagnose with atypical RICH combined with VM [6].

Treatment choice is difficult for this rare case. En bloc resection of the tumor may be the best treatment to prevent recurrence. But due to the nerves, tendon, ligament, and essential structures surrounding the tumor, it will be easy to cause the physical disability if the tumor is excised completely. Patient in this case involved all tissues and growth is progressive after partial resections. The main purpose of the surgery is to release the local symptoms such as persistent pain and numbness, increasing size of the mass, and functional impairment. The patient in this case had several aggressive surgeries, which not only stimulated the growth of the tumor but also resulted in unnecessary injuries in other parts such as the donor site. Therefore, it needs to avoid similar crisis in the future. A prosthesis was fitted after the amputation, the patient could raise his shoulder, and his ability of self-care was obviously improved. Sclerotherapy, chemoembolization, and embolotherapy [14] are treatment options for very vascularized tumors or arterio-venous malformations. Currently, therapy with Sirolimus is considered in severe cases with enhancing results.

## Conclusions

For this rare tumor with repeated recurrences and significant impact on the daily life, we suggest performing an amputation at the early stage.

## Abbreviations

RICH: Rapidly involuting congenital hemangioma
LM-VM: Lymphatic and venous
CLVM: Capillary-lymphatic-venous malformation
ESR: Erythrocyte sedimentation rate
